# Reduced-Complexity Cultivation of Human Oral Anaerobic Bacteria Using a Microdroplet-Based Culturomics Platform

**DOI:** 10.4014/jmb.2607.07042

**Published:** 2026-10-08

**Authors:** Chaeeun Kang, You-Jeong Kim, Min-gyung Baek, Aram J. Chung, Hana Yi

**Affiliations:** 1 Integrated Biomedical and Life Science, Graduate School, Korea University, Seoul 02841, Republic of Korea; 2 Interdisciplinary Program in Precision Public Health, Korea University, Seoul 02841, Republic of Korea; 3 Department of Bioengineering, Korea University, Seoul 02841, Republic of Korea; 4 Institute for Biomaterials, Korea University, Seoul 02841, Republic of Korea; 5 School of Biomedical Engineering, Korea University, Seoul 02841, Republic of Korea; 6 School of Biosystems and Biomedical Sciences, Korea University, Seoul 02841, Republic of Korea

**Keywords:** Microdroplet microfluidics, Culturomics, Human oral microbiota, Fastidious anaerobes, Reduced-complexity cultivation

## Abstract

The human oral microbiota contains fastidious anaerobes that remain uncultivated due to resource competition and overgrowth by dominant species in conventional plating. Culturomics approaches leveraging droplet-based microfluidics offer an alternative by physically compartmentalizing microbial cells. We established a polydimethylsiloxane-based microdroplet platform to isolate anaerobic bacteria from human oral rinse samples. Droplet generation conditions were optimized using fluorescent beads, and bacterial loading at the collection-drop level was evaluated using *Escherichia coli*. Two downstream isolation strategies—Direct-plating (Method 1) and Enrichment (Method 2)—were compared against conventional serial dilution across various media compositions. Isolated strains were identified via 16S rRNA gene sequencing. At optimized flow rates (oil: 1.5 mL/h, core: 0.3 mL/h), the system produced uniform microdroplets with an average occupancy (λ) of 0.010–0.012 cells/droplet, corresponding to a high theoretical probability of single-cell occupancy among non-empty microdroplets. Each macro-collection drop delivered approximately 50-60 cells into recipient wells, serving as a reduced-complexity cultivation unit. Under the matched solid-medium condition, Method 1 achieved the highest taxonomic yield (14.3%), representing a 3.5-fold enhancement over conventional serial dilution (4.1%). Method 2 yielded 4.7%, indicating that liquid enrichment reintroduced growth bias toward fast-growing taxa. Among the tested media, unsupplemented Tryptic Soy Agar preserved the highest species diversity, whereas 5% sheep blood supplementation led to monoculture dominance by fast-growing taxa. Overall, the platform isolated diverse oral anaerobic species including fastidious taxa like *Capnocytophaga*, *Cardiobacterium*, and *Eikenella*. The microdroplet-based reduced-complexity platform may limit the dominance of rapidly growing taxa, providing a complementary culturomics tool for expanding human oral anaerobic bacterial resources.

## Introduction

The human oral cavity is a complex ecosystem harboring distinct microbial communities shaped by unique microenvironments, including teeth surfaces, subgingival niches, tongue, and saliva. The human oral microbiota has gained significant clinical and biological attention as its intimate associations not only with localized oral diseases, such as dental caries and periodontal diseases, but also with systemic inflammatory disorders have been progressively uncovered. However, elucidating the metabolic functions of oral microbial ecosystems and their pathobiological interactions with the host requires a “culturomics” approach—going beyond next-generation sequencing (NGS)-based community analysis to pure-isolate actual living bacterial resources and obtain high-quality reference genomes. The recently established Cultivated Oral Bacteria Genome Reference (COGR) database also strongly highlights the critical need to expand oral bacterial resources through large-scale isolation and cultivation [1].

Nevertheless, among the more than 700 bacterial species known to inhabit the oral cavity, a substantial proportion remains unrecovered by conventional agar plate-based cultivation methods. In particular, anaerobic bacteria, extremely slow-growing species, or fastidious uncultivated strains with complex nutritional requirements easily perish or are omitted under standard solid media conditions. According to previous studies, the per-cell recovery of conventional plating in oral microbial isolation is merely around 1%, and alternative approaches based on single-cell cultivation have been reported to overcome this bottleneck [2]. In traditional serial dilution and spread-plating, dominant species within a sample pre-occupy ecological niches and proliferate rapidly, posing an inherent limitation where rare taxa present at low abundances or with slow growth rates are outcompeted during colony formation. Indeed, streamlined cultivation studies targeting the human oral microbiota demonstrated an exclusive bias, repeatedly recovering *Streptococcus* species at a disproportionately high frequency [3].

To overcome these physical and biological constraints, “dilution-to-extinction cultivation”—which isolates complex microbial samples down to the single-cell level for extended culture—has emerged as a viable alternative [4-6]. Recently, “droplet-based microfluidics” integrating microfluidic control technology has gained significant attention as an innovative platform to execute this process at high speed and throughput. By exploiting the interfacial properties of immiscible oil and aqueous phases, this technology generates highly uniform water-in-oil droplets and utilizes individual droplets as independent micro-bioreactors [7, 8]. This provides powerful advantages, including ultra-high throughput and minimal reagent consumption, significantly enhancing the recovery rate of fastidious anaerobic strains in gut microbiome studies [9].

In this study, to efficiently convert uncultivated human oral anaerobic microorganisms into biological resources, we established a microdroplet-based cultivation platform. We systematically validated its hydrodynamic operational conditions, encapsulation efficiency, bacterial viability following oil exposure, and real-world applicability to human oral rinse samples. Through these efforts, we aim to demonstrate that this platform serves as a complementary cultivation strategy that maximizes the recovery of oral microbial diversity relative to limited colony-picking efforts.

## Materials and Methods

### Construction of the Microdroplet Cultivation Platform

A polydimethylsiloxane (PDMS)-based microfluidic chip was fabricated using standard soft lithography for the isolation and cultivation of human oral anaerobic bacteria (Fig. 1A). Briefly, a microchannel mold was prepared by conventional photolithography using SU-8 photoresist (MicroChem Corp., USA), and PDMS (Sylgard 184, Dow Corning, USA) was cast onto the mold to replicate the microchannel structures. Inlet and outlet ports were created using a biopsy punch, after which the patterned PDMS layer was irreversibly bonded to a glass slide by oxygen plasma treatment (CUTE, Femto Science, Republic of Korea). The bonded devices were subsequently baked at 75°C for at least 24 h to enhance bond strength and ensure stable device performance.

The completed chip consisted of a single oil inlet, a single core inlet, and a single outlet, with a fixed channel height of 20 μm (Fig. 1B). To minimize channel clogging caused by particulate debris or bacterial aggregates, integrated microfilter structures were positioned upstream of both inlet channels. QX200 Droplet Generation Oil for EvaGreen (Bio-Rad, USA) was used as the continuous oil phase, whereas microbial culture suspensions or microbial suspensions prepared from human mouthwash samples were introduced through the core inlet as the dispersed aqueous phase. Prior to use, the oil was filtered through both 0.2 μm hydrophilic and hydrophobic polytetrafluoroethylene (PTFE) syringe filters. All microdroplet generation and cultivation procedures were performed inside a COY anaerobic chamber (Coy Laboratory Products, USA) under strictly anaerobic conditions (Fig. 1C).

### Optimization of Droplet Generation Using Fluorescent Beads

To optimize fluidic operating conditions and quantitatively estimate the effective cell count within the final collection drop, fluorescent beads with a diameter of 9.9 μm were used as microbial cell surrogates. To avoid terminology confusion in this paper, micro-sized droplets generated continuously inside the chip channels were defined as “microdroplets,” whereas macroscopic drops collected at the outlet were defined as “collection drops”.

The bead suspension was diluted 10^−2^ and introduced into the core inlet. Microdroplet generation behavior was monitored while incrementally adjusting the flow rate ratio between the oil and core phases. Multiple oil-to-core flow rate combinations were empirically tested, and the final operating condition was selected based on stable and continuous microdroplet generation without obvious breakage or coalescence, together with successful downstream bacterial recovery. The collection drop generation period at the outlet, the internal microdroplet generation rate, and the bead discharge frequency were quantitatively analyzed using a high-speed camera (Phantom VEO 701L, Vision Research, USA).

Based on this, the number of microdroplets and beads co-encapsulated per collection drop was estimated. The final operational parameters were applied to actual sample conditions to calculate the expected bacterial cell count per collection drop. Furthermore, based on the calculated cell density, the average cell occupancy (λ) per microdroplet was derived to evaluate single-cell encapsulation conditions from a Poisson distribution perspective.

### Validation of Bacterial Loading at the Collection-Drop Level

To evaluate bacterial loading at the collection-drop level under the optimized fluidic conditions, *Escherichia coli* was selected as a model strain. Droplets were generated using a cell culture diluted 10^−3^. Collected collection drops were suspended in 1 mL of nuclease-free water and stained with DAPI (4',6-diamidino-2-phenylindole). The stained cell suspension was filtered under reduced pressure through a 0.2 μm black-stained polycarbonate membrane and observed/counted under a fluorescence microscope. To evaluate temporal reproducibility, collection drops were collected at 10-min intervals across Early, Mid, and Late stages of the process. Total cell counts across 10 collection drops were measured per time point to derive the mean and standard deviation. A total of 30 measurements were obtained. Measurements with zero detected cells were retained as valid observations. One measurement in which the cell density was too high for reliable enumeration was excluded because an exact numerical count could not be assigned.

### Evaluation of Bacterial Viability Following Oil Exposure

To assess whether the oil used for droplet generation exerts toxicity on bacterial survival, an oil exposure assay was conducted using *E. coli*. Cell suspensions diluted at 10^−1^ and 10^−2^ were mixed with nuclease-free water, droplet generation oil, baby oil, and cooking oil, respectively. The mixtures were plated on Luria-Bertani (LB) agar and incubated under anaerobic conditions at 37°C. Following incubation, colony formation patterns were analyzed to evaluate viability, and the mixing behavior and media absorption characteristics of each oil were compared.

### Microdroplet-Based Isolation of Oral Bacteria

To evaluate the applicability of the microdroplet-based cultivation platform to clinical oral samples, isolation was performed using human oral rinse samples. All oral rinse samples used in this study were obtained from a single donor. Tryptic Soy Agar (TSA; BD Difco, USA) was selected as a broadly supportive basal medium for evaluating bacterial recovery under different supplementation conditions, and conditions supplemented with bacitracin (50 mg/L), surfactants (sodium oleate 0.15% and sodium lauryl sulfate 0.01%), and 5% sheep blood were systematically compared.

Individual collection drops discharging from the outlet were collected—one drop per well—into 96-well plates containing 100 μL of Tryptic Soy Broth (TSB; BD Difco, USA). TSA/TSB was used as a standardized basal medium rather than a taxon-specific medium to allow comparison of the effects of the cultivation workflow and defined supplements. To obtain pure single colonies, two downstream strategies were evaluated:

Direct-plating method (Method 1): In this method, TSB was utilized solely as a carrier medium to physically collect droplets and maintain spatial isolation between wells, rather than as a growth medium. Without prior enrichment, the entire 100 μL suspension from each well was homogenized by pipetting and immediately spread onto solid media. Plated media were incubated anaerobically at 37°C. By eliminating intra-well incubation time, this strategy directly captures colonies using only physical compartmentalization.

Enrichment method (Method 2): The initial step of collecting droplets into 96-well plates containing 100 μL TSB was identical to Method 1. However, instead of immediate plating, the 96-well plates were pre-incubated anaerobically at 37°C to induce bacterial growth within individual wells. Turbidity (OD_600_) was measured daily during the first week and weekly thereafter up to 1 month. Wells exhibiting an OD_600_ ≥ 0.5 were considered positive for growth, diluted 10^−2^ with nuclease-free water, and 100 μL was spread onto solid media for anaerobic incubation at 37°C. This strategy was designed to improve recovery efficiency by allowing low-density encapsulated cells time to proliferate to detectable levels.

The 96-well plates were maintained with their standard lids in place and incubated at 37°C inside an anaerobic jar containing a GENbox anaerobic gas pack (bioMérieux, France). This enclosed configuration was maintained to minimize evaporation during prolonged incubation. No additional TSB was supplied during the 1-week to 1-month incubation period.

Single colonies were selected for subsequent purification. When colony numbers were high, morphologically distinguishable colonies were preferentially selected to maximize the taxonomic breadth of recovered isolates; colony morphology itself was not used for taxonomic assignment. The selected colonies were subcultured iteratively on fresh solid media under anaerobic conditions at 37°C to obtain pure cultures.

Final isolates were identified by amplification of the 16S rRNA gene using the universal bacterial primers 27F (5′-AGAGTTTGATCMTGGCTCAG-3′) and 1492R (5′-TACGGYTACCTTGTTACGACTT-3′). PCR amplification was performed with an initial denaturation at 95°C for 5 min, followed by 25 cycles of denaturation at 95°C for 30 s, annealing at 56°C for 30 s, and extension at 72°C for 90 s, with a final extension at 72°C for 10 min. PCR amplicons were subjected to Sanger sequencing using capillary electrophoresis sequencing. The resulting 16S rRNA gene sequences were compared against the EzBioCloud database for taxonomic identification.

To compare bacterial recovery efficiency among the different isolation methods, expected species richness was calculated from the abundance of identified bacterial species using the rarefy function in the vegan package (version 2.6-4) implemented in R. This analysis was used to standardize differences in isolate-screening depth and was not intended to normalize the different incubation histories of Methods 1 and 2.

## Results

### Establishment of Microdroplet Platform and Optimization of Fluidic Operation

Screening across various flow rate combinations revealed that a uniform stream of microdroplets was continuously generated without droplet breakage or coalescence at an oil flow rate of 1.5 mL/h and a core flow rate of 0.3 mL/h. Quantitative predictions using fluorescent beads (under oil 1.5 mL/h, core 0.05 mL/h) showed that collection drops were discharged at the outlet approximately every 5 s. High-speed video analysis indicated a microdroplet generation frequency of approximately 1,000 microdroplets/s. Hydrodynamic calculations estimated that each single collection drop contained approximately 5,000 microdroplets and 100 beads.

Translating these parameters to our final optimized operating conditions (core flow rate 0.3 mL/h) and sample dilution factor (10^−3^), each collection drop was predicted to harbor approximately 50−60 bacterial cells evenly distributed and encapsulated. Based on this, the average cell occupancy (λ) per microdroplet was calculated to be 0.010–0.012 cells/microdroplet. From a Poisson loading perspective, such an extremely low value indicates that while the vast majority of microdroplets remain empty, non-empty microdroplets have a maximized probability of containing single cells. At λ = 0.010–0.012, approximately 99.4–99.5% of non-empty microdroplets are theoretically expected to contain a single cell; however, individual cell occupancy within microdroplets was not directly visualized in this study. However, because our platform collects entire macro-collection drops containing thousands of microdroplets into individual wells of 96-well plates rather than culturing microdroplets in complete isolation, the final culture unit is appropriately interpreted as a “reduced-complexity cultivation unit” rather than a strict single-cell culture.

### Evaluation of Bacterial Loading Reproducibility and Viability Following Oil Exposure

Using *Escherichia coli* as a model strain, temporal reproducibility of bacterial loading at the collection-drop level during microfluidic operation was evaluated. Mean cell counts per collection drop retrieved across process timepoints were 44.6 ± 49.9 (Early), 36.2 ± 46.5 (Mid), and 39.6 ± 47.0 (Late). Despite variability among individual collection drops, mean bacterial loading remained broadly comparable across the sampling time points. The relatively large standard deviations relative to the mean are consistent with the expected behavior of a low-occupancy Poisson loading process (λ = 0.010–0.012), in which bacterial cells are stochastically distributed among a large number of microdroplets prior to collection; this inherent stochasticity, rather than inconsistent device performance, is considered the primary source of the observed drop-to-drop variability.

Oil exposure assays evaluating potential oil toxicity on bacterial viability demonstrated a minor reduction in cell density across all three tested oils compared to the control (nuclease-free water). At high cell concentrations (10^−1^ dilution), colony counts were comparable between control and oil-treated groups. However, at lower concentrations (10^−2 ^dilution), reductions in colony-forming units (CFUs) relative to the control were 15% for droplet generation oil, 18% for baby oil, and 20% for cooking oil. Notably, commercial cooking oil and baby oil remained on the surface of solid media, physically obstructing bacterial gas exchange and colony formation. In contrast, droplet generation oil exhibited excellent interfacial compatibility with aqueous suspensions and absorbed rapidly into the agar matrix without hindering bacterial growth, proving to be the most suitable carrier oil for downstream cultivation.

### Effect of the Enrichment Step on Bacterial Recovery

We evaluated the impact of an enrichment step on bacterial recovery rate and diversity when culturing bacteria from recovered collection drops. We compared immediate spread-plating without prior liquid culture (Direct-plating, Method 1) against liquid enrichment in 96-well plates prior to agar plating (Enrichment, Method 2) (Fig. 2).

Out of 192 total collection drop-inoculated wells plated onto 192 agar plates in Method 1, colony growth was observed on 28 plates, yielding 49 single colonies representing 7 distinct bacterial species. In contrast, Method 2 exhibited positive growth in 73 of 192 wells, yielding 128 single colonies representing 6 bacterial species.

Clear differences were also observed in dominant species composition between the two strategies. Method 1 yielded high proportions of *Veillonella rogosae* and *Veillonella hominis*, along with *Staphylococcus epidermidis*, *Capnocytophaga* sp., and *Cardiobacterium hominis*. Conversely, Method 2 was heavily dominated by *Veillonella dispar*, *Fusobacterium hwasookii*, and *Fusobacterium polymorphum*, alongside *Eikenella corrodens* (Supplementary Table S1, Fig. 3A).

### Comparison of Oral Bacterial Isolation Efficiency with Conventional Serial Dilution

To evaluate the performance of the microdroplet-based cultivation platform, bacterial recovery was compared with conventional serial dilution using the same final solid-medium formulation and anaerobic plating condition. For this comparison, Method 1, Method 2, and the conventional method were all evaluated using TSA supplemented with sodium oleate, sodium lauryl sulfate, bacitracin, and 5% sheep blood; Method 2 additionally included the liquid enrichment step described above. For the conventional method, all colonies formed on the 10^-2^ dilution plate were isolated and identified via 16S rRNA gene sequencing.

The conventional serial dilution method yielded only 4 bacterial species from 97 isolates. In contrast, the microdroplet platform recovered 7 species from 49 isolates using the Direct-plating strategy (Method 1) and 6 species from 128 isolates using the Enrichment strategy (Method 2) (Table 1). Notably, Method 1 recovered the highest number of species despite requiring roughly half the number of screened isolates compared to the conventional method.

Isolate-based rarefaction analysis further supported the higher taxonomic yield observed for Method 1 at comparable screening depths. Across comparable screening depths, Method 1 consistently exhibited higher expected species richness than either Method 2 or the conventional serial dilution method, indicating more efficient taxonomic recovery per screened isolate. This rarefaction analysis accounts for differences in isolate-screening depth but not for the different cultivation histories of Methods 1 and 2.

The taxonomic yield per screened isolate, calculated as the number of recovered species divided by the total number of screened isolates, was 14.3% for Method 1, compared with 4.7% for Method 2 and 4.1% for the conventional serial dilution method (Table 1). Consequently, applying Method 1 achieved approximately a 3.5-fold higher taxonomic yield per screened isolate than the conventional method. Detailed bacterial strain compositions and lists of recovered isolates for each strategy are provided in Supplementary Table 1.

### Comparison of Oral Bacterial Recovery according to Culture Media Composition

To assess the effect of media composition, Method 2 was fixed while solid media supplements were varied. Basal TSA alone yielded the highest taxonomic diversity (14 genera and species, including *Actinomyces*, *Streptococcus*, *Parvimonas*, *Schaalia*, and *Veillonella*) from 34 colonies (Table 1, Supplementary Table S1, Fig. 3B). Addition of selective agents and surfactants (sodium oleate, sodium lauryl sulfate, and bacitracin) slightly reduced identified species to 12 (including *Campylobacter concisus* and *Fusobacterium* spp.). Further supplementation with 5% sheep blood drastically increased total recovered colonies to 128, but species diversity plummeted to 6 species. This was attributed to explosive overgrowth of *Veillonella dispar* and *Fusobacterium* strains on nutrient-rich media, which monopolized the plates.

Across positive wells, the mean number of species identified per well was 1.53. Out of 160 valid wells, 88 wells yielded a single bacterial strain, while several wells contained 2−4 species due to nutrient availability and co-growth (Supplementary Table S2). Through microdroplet-based system optimization and screening, a total of 278 purified isolates were obtained. Together with 97 isolates obtained by conventional serial dilution, 375 isolates were analyzed across the entire study, representing 29 distinct oral bacterial species identified via 16S rRNA gene sequencing (Supplementary Table S3).

## Discussion

In this study, to efficiently convert fastidious microbial strains from the complex human oral anaerobic microbiota into biological resources, we established a microdroplet-based cultivation platform and validated its utility by applying it to actual human oral rinse samples. By partitioning samples into numerous finely controlled microdroplets and collection drop units, this platform was designed to reduce the initial microbial complexity entering individual downstream cultivation units, thereby potentially limiting dominance by rapidly growing taxa—an inherent limitation of conventional agar plating. Under optimal operating conditions, approximately 50−60 cells were predicted to be uniformly distributed per collection drop, and temporal DAPI fluorescence counting was used to evaluate bacterial loading at the collection-drop level across the sampling time points. These results demonstrate that beyond the limitations of simple single-cell isolation, this system successfully operates as a reduced-complexity cultivation platform that maximizes cultivation efficiency by step-wise mitigating the complexity of multi-species microbial samples.

In droplet-based microfluidics, cell encapsulation is generally interpreted based on Poisson statistics. Collins *et al*. [10] reported that passive droplet encapsulation is constrained by a Poisson distribution, where single-cell encapsulation efficiency is determined by droplet volume and cell concentration. Lu *et al*. [11] also confirmed that cell encapsulation follows a Poisson distribution, showing that when the average cell concentration is ≤0.1 cells/droplet, single-cell droplets account for over 95% of all non-empty droplets. The value derived in our study (λ = 0.010−0.012 cells/microdroplet) is well below this theoretical benchmark, representing a condition that maximizes the probability of encapsulated cells existing as single cells at the microdroplet level. However, it is appropriate to interpret the low λ value obtained here not merely as a choice aimed strictly at single-cell isolation itself, but rather as a device-specific operational condition derived by considering an ecological balance between minimal microdroplet occupancy and practical bacterial recovery. Indeed, when the core flow rate was increased from 0.05 mL/h to 0.3 mL/h, the expected number of cells per collection drop was secured at approximately 50−60 cells, which finally enabled meaningful bacterial recovery. This demonstrates that maintaining low microdroplet occupancy to minimize multiple-cell occupancy while effectively securing a cultivable number of bacterial cells was a more fundamental factor in operating this platform than unconditionally minimizing empty droplets.

This flow rate optimization approach aligns well with trends in previous studies dealing with microbial droplet cultivation. Flow rate parameters in droplet-based cultivation platforms are not universal fixed values; rather, they have been empirically established in various ways depending on chip geometry, droplet size, cell density, oil-to-aqueous flow rate ratio, and downstream cultivation strategy. For instance, Dong *et al*. [12] set the flow rates of cell suspension, media, and carrier oil to 0.25, 0.3, and 3.0 μL/min, respectively, applying an oil-to-aqueous flow rate ratio of approximately 5.5:1 during single-cell bacterial culture. Zhang *et al*. [13] adopted a higher ratio using 0.5 μL/min cell suspension and 6 μL/min mineral oil. Conversely, Staskiewicz *et al*. [14] applied a relatively lower flow rate ratio (1.67:1) using 500 μL/h oil and 300 μL/h aqueous phase for droplet culture of biosurfactant-producing bacteria. This suggests that rather than a single universal flow rate existing for microdroplet-based bacterial culture, fine-tuning fluidic parameters according to each platform's purpose and chip architecture is standard practice. The final condition chosen in this study (1.5 mL/h oil flow rate and 0.3 mL/h core flow rate, 5:1 oil-to-core ratio) validly falls within the range verified by prior literature, supporting its selection as a practical hydrodynamic operating condition that provided both stable droplet generation and practical bacterial recovery within our microfluidic chip.

The reduced-complexity approach presented in this study holds major significance in oral ecosystems, where extreme disparities exist in bacterial growth rates and media adaptability. In conventional agar plating, dominant species with rapid proliferation rates or superior media adaptability (*e.g.*, *Streptococcus* spp.) monopolize spatial niches and nutrients across the entire plate, thereby inhibiting colony formation by rare taxa that grow slowly or exist at low abundances. In this study, by physically compartmentalizing ecological spaces between microbes into collection drop units, the mean number of identified species per positive well in 96-well plates was markedly controlled to 1.53 species. This indicates that the initial taxonomic complexity of individual cultivation units was substantially reduced, which may have limited opportunities for competitive dominance during downstream recovery. This aligns with previous findings that droplet-based anaerobic culture enhances taxonomic richness and favors the recovery of rare strains [8, 15].

Comparative analysis with the conventional serial dilution method revealed distinct bacterial recovery patterns depending on the downstream recovery strategy applied to our microdroplet platform. The taxonomic yield per screened isolate was 4.1% for the conventional serial dilution method and slightly increased to 4.7% for the microdroplet-based Enrichment strategy (Method 2), showing no significant difference from the conventional method. On the other hand, the microdroplet-based Direct-plating strategy (Method 1) achieved a high taxonomic yield per screened isolate of 14.3%, representing an approximate 3.5-fold enhancement over the conventional culture method (Table 1). This trend was further corroborated by isolate-based rarefaction analysis, which demonstrated that Method 1 consistently achieved higher expected species richness than either Method 2 or the conventional serial dilution method across comparable screening depths. These results indicate that the higher expected species richness observed for Method 1 was not solely attributable to differences in the number of screened isolates. Together, these findings demonstrate that micro-spatial compartmentalization can facilitate taxonomic recovery under limited colony-screening efforts.

Simultaneously, however, they highlight that final recovery efficiency relies heavily on whether post-collection liquid enrichment is performed. Even when physical cell separation is successfully achieved at the collection drop level, subsequent liquid enrichment within recipient wells allows specific fast-growing taxa with high media adaptability to regain competitive dominance. Consequently, the ecological species composition bias typical of conventional agar plating is re-introduced. Therefore, maximizing the performance of a microdroplet-based cultivation platform requires comprehensive optimization of not only the fluidic droplet generation process, but also the downstream recovery strategy.

Comparative results according to sample processing and screening strategies confirmed that the Direct-plating method (Method 1) and the Enrichment method (Method 2) provide distinct ecological selective pressures. Method 1 eliminated the liquid growth phase and directly spread collection drops onto solid media, preventing fast-growing bacteria from having the temporal opportunity to dominate within wells. Consequently, while the total number of positive wells (28/192 wells) and colonies (49 colonies) was lower, fastidious taxa easily masked by dominant species in standard plating—such as *Capnocytophaga* sp. and *Cardiobacterium hominis*—were successfully recovered. In contrast, Method 2 significantly improved colony recovery efficiency via preliminary liquid culture (73/192 wells, 128 colonies), but led to a skewed dominance of *Veillonella dispar*, *Fusobacterium hwasookii*, and *Fusobacterium polymorphum*, which adapt exceptionally well to liquid media environments. These findings support the complementary application of both Direct-plating and Enrichment strategies rather than depending on a single isolation technique [2]. However, because Methods 1 and 2 differ in cultivation history, these results should be interpreted as differences between complete downstream recovery workflows rather than as a strictly controlled comparison of intrinsic cultivation efficiency.

Differences stemming from media supplements also provided clear ecological guidelines. Under plain TSA basal medium conditions, single-strain overgrowth was suppressed, securing the highest species diversity (14 species). Conversely, under conditions supplemented with 5% sheep blood, total colony count increased drastically, but final identified species plummeted to 6 species due to the monopoly of specific *Veillonella* and *Fusobacterium* species. This shows that adding nutrients or selective agents does not necessarily lead to an increase in recovered species richness, but can instead trigger explosive growth of specific dominant species, thereby reducing overall isolation diversity.

In particular, strains recovered through this platform, such as *Capnocytophaga* sp., *Cardiobacterium hominis*, and *Eikenella corrodens*, carry major clinical and ecological significance. *Capnocytophaga* is a slow-growing strain present in dental plaque [16], while *Cardiobacterium hominis* and *E. corrodens* are fastidious resident members of the HACEK (*Haemophilus*, *Aggregatibacter*, *Cardiobacterium*, *Eikenella*, and *Kingella*) group that require days to weeks for clinical cultivation [17, 18]. The pure isolation of these strains—which are easily outcompeted and eliminated by dominant species in conventional plating—supports the practical utility of microdroplet-based reduced-complexity cultivation for recovering fastidious oral bacteria, although the present study does not directly establish reduced interspecies competition as the underlying mechanism.

Meanwhile, results from the oil exposure assay demonstrate both the potential and limitations regarding survival rates and process compatibility. All three tested oils induced a 15−20% CFU reduction compared to the water control group, revealing the potential for reduced viability upon oil exposure. However, unlike cooking oil or baby oil, which remained on the agar surface and blocked gas exchange, droplet generation oil exhibited superior miscibility with aqueous suspensions and excellent media absorption properties, making it the most suitable process carrier oil.

Limitations of this study and future tasks are as follows: First, the λ values presented in this study were calculated based on bead-based estimations and final sample dilution factors, which do not fully reflect cell aggregation, cell viability, culturability, or microdroplet size variations in actual oral clinical samples. Second, additional fine control of microfluidic flow is required to reduce cell count variance between collection drops. Third, comparisons of media conditions and recovery methods were conducted under select conditions rather than evaluating all condition combinations through a full factorial design. In addition, Method 1 and Method 2 differed in their downstream cultivation histories; therefore, their comparison reflects the performance of complete recovery workflows rather than a strictly controlled comparison of intrinsic cultivation efficiency. Because this workflow-level comparison was performed using a single sample type and a single set of media conditions, the magnitude and direction of this bias may differ when the platform is applied to other sample types, such as environmental or other clinical specimens with distinct microbial community structures and growth kinetics. Further studies across diverse sample types will therefore be required to determine how broadly the observed trends generalize. Finally, although isolates were identified by 16S rRNA gene sequencing, morphology-guided colony selection may have undersampled phenotypically similar but taxonomically distinct colonies, and the limited TSA-based media evaluated here may not support all fastidious oral taxa. Furthermore, reduced interspecies competition was not directly measured in this study and should therefore be considered a potential explanation rather than an experimentally established mechanism.

In conclusion, rather than completely replacing traditional plate cultivation, the microdroplet-based cultivation platform established in this study serves as a powerful complementary culturomics tool that efficiently secures uncollected anaerobic microbial resources within the oral ecosystem by combining spatial separation technology with condition-specific screening strategies.

## Supplemental Materials

Supplementary data for this paper are available on-line only at http://jmb.or.kr.



## Figures and Tables

**Fig. 1:**
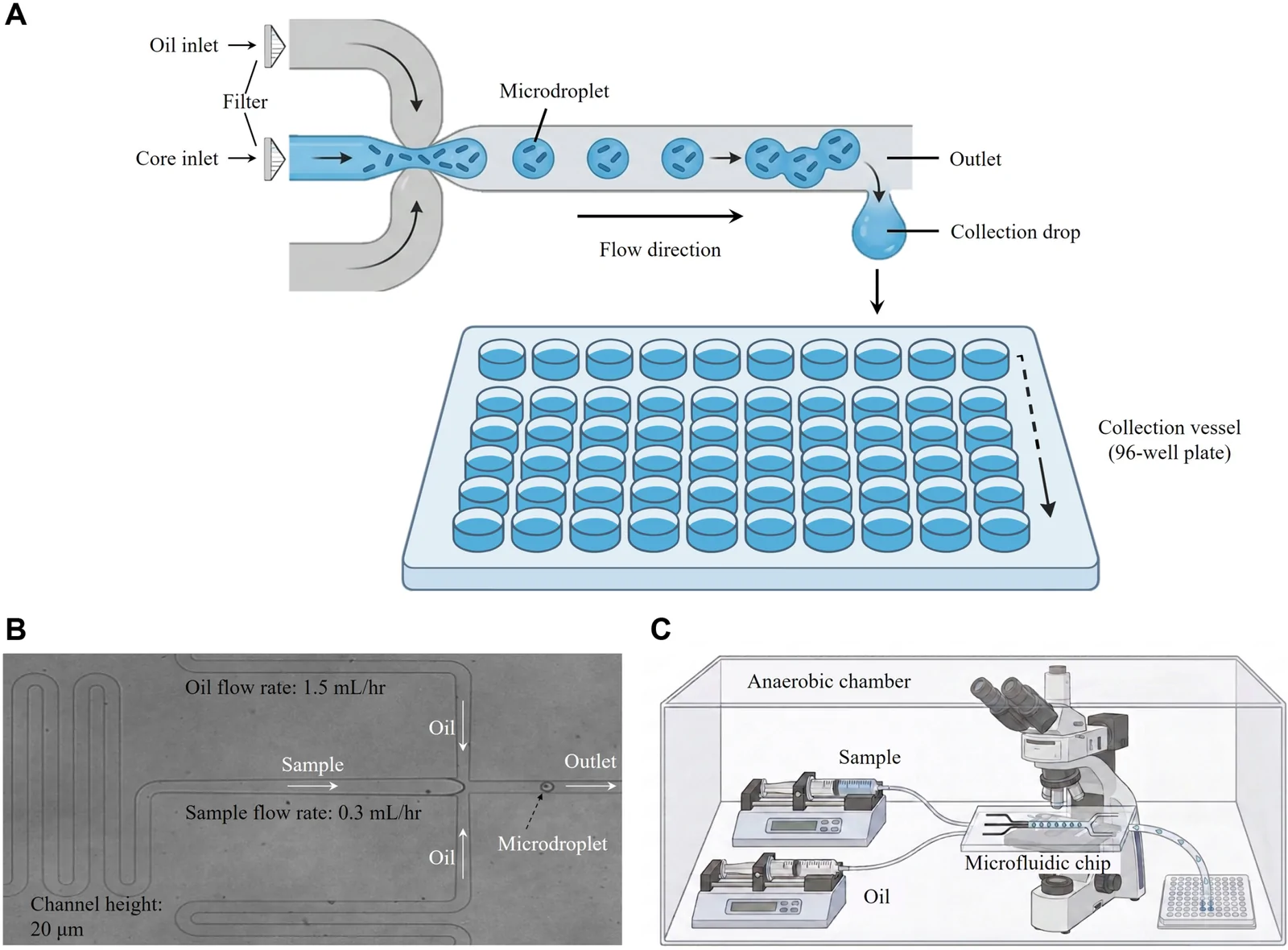
Overview and operation of the microdroplet-based platform for oral anaerobic bacterial isolation. (**A**) Schematic diagram illustrating microdroplet generation via flow focusing with integrated inlet filters, followed by the discharge of macro-collection drops into individual wells of a 96-well plate. (**B**) Optical micrograph of the PDMS microfluidic chip during operation (channel height: 20 μm), displaying optimized flow conditions for oil (1.5 mL/h) and core sample (0.3 mL/h) phases. (**C**) Schematic representation of the complete experimental setup, including syringe pumps, microfluidic chip, and microscope, configured inside an anaerobic chamber to ensure strictly anaerobic cultivation conditions.

**Fig. 2:**
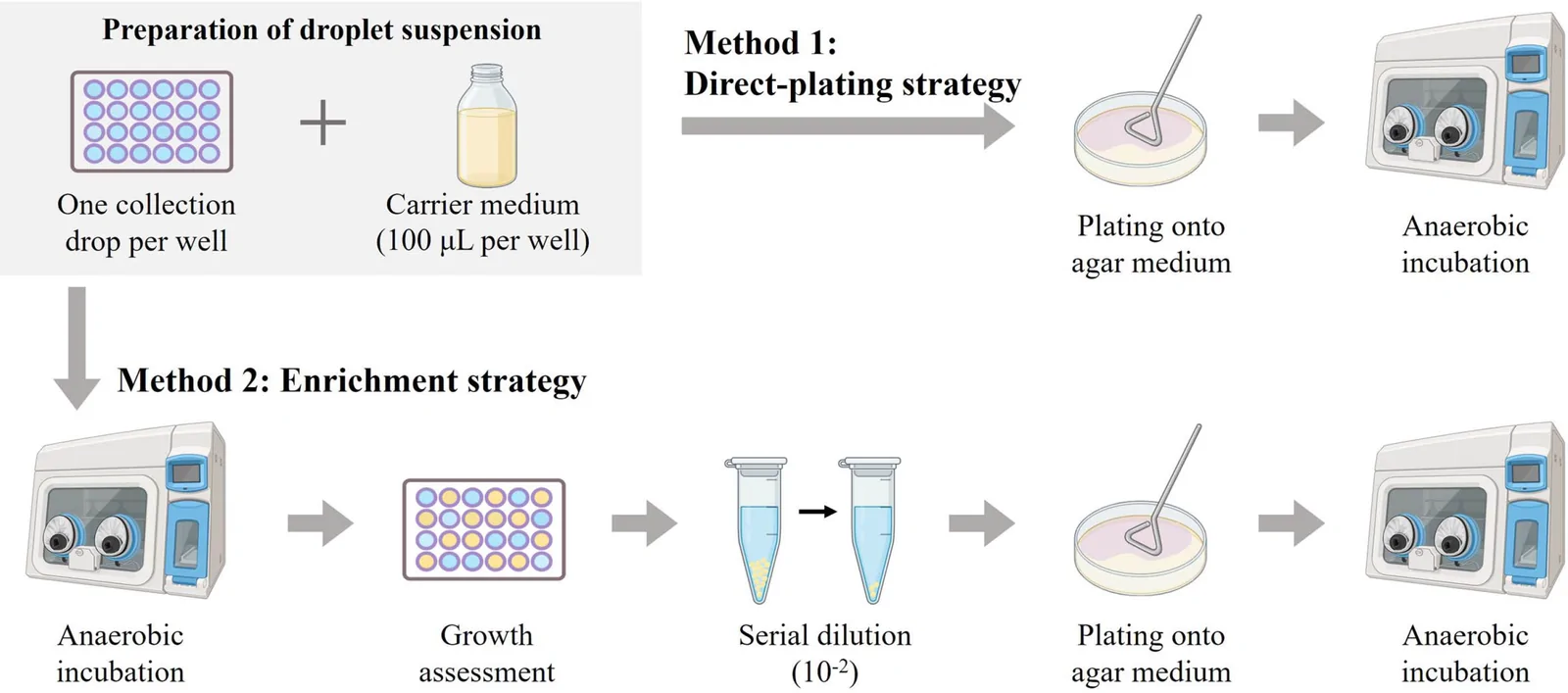
Schematic workflow of downstream recovery strategies using the microdroplet platform. Macro-collection drops are individually dispensed into a 96-well plate containing 100 μL of liquid carrier medium (Tryptic Soy Broth) per well to prepare the initial droplet suspension. In Method 1 (Direct-plating strategy), the homogenised suspension from each well is directly spread onto solid agar medium without prior liquid incubation, followed by anaerobic culture. In Method 2 (Enrichment strategy), recipient plates undergo primary liquid incubation under anaerobic conditions; positive growth wells (OD_600_ ≥0.5) are subsequently diluted (10^−2^) and plated onto solid agar medium for final isolate recovery.

**Fig. 3:**
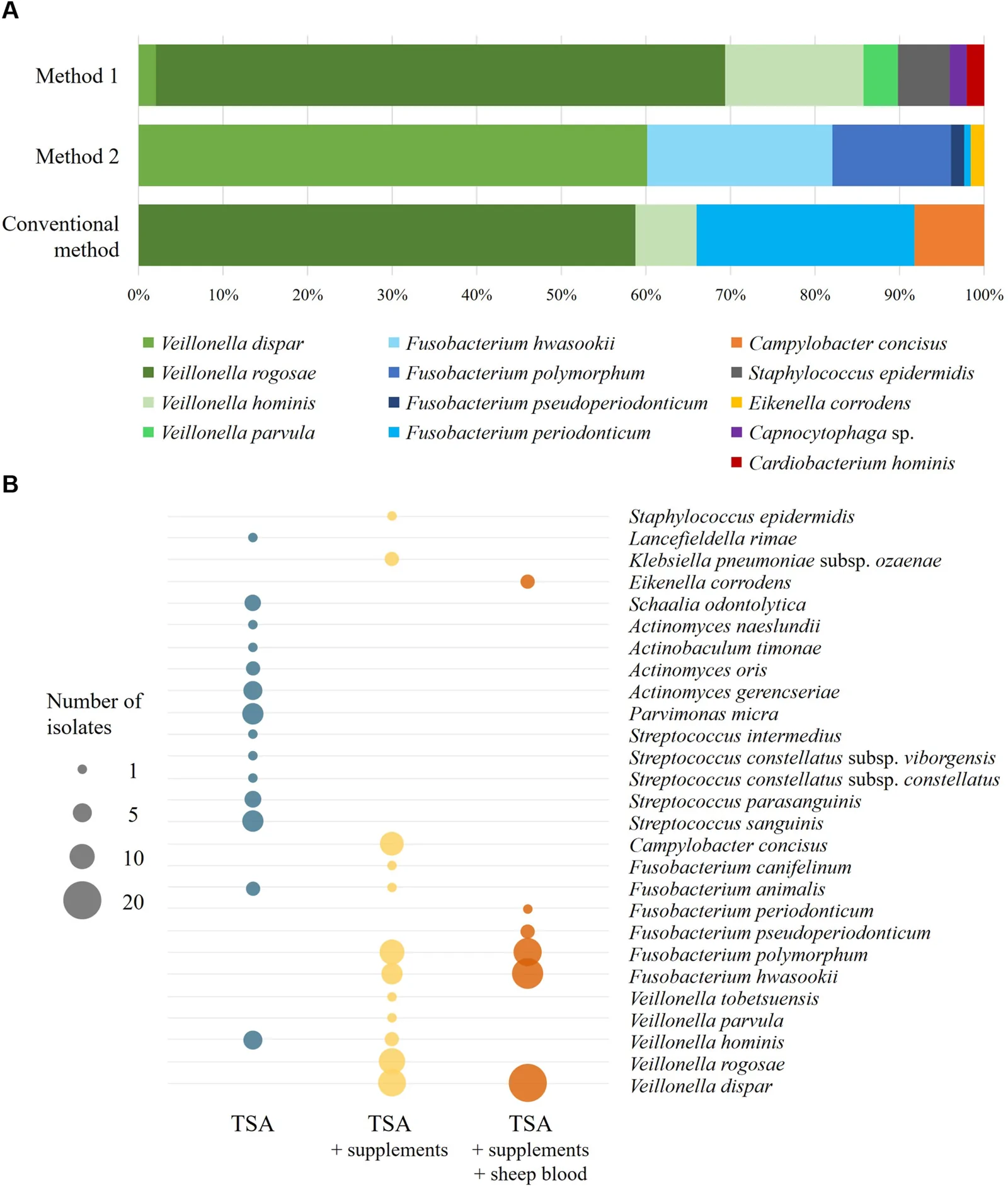
Taxonomic composition of bacterial isolates across recovery strategies and culture media conditions. (**A**) Relative abundance and species-level composition of bacterial isolates recovered using the direct-plating strategy (Method 1), enrichment strategy (Method 2), and conventional serial dilution. For this workflow comparison, all three strategies were evaluated using the same final solid-medium formulation consisting of TSA supplemented with sodium oleate (0.15%), sodium lauryl sulfate (0.01%), bacitracin (50 mg/L), and 5% sheep blood; Method 2 additionally included liquid TSB enrichment before plating. (**B**) Distribution and isolate counts of 27 bacterial species recovered under different solid-medium compositions evaluated using Method 2. Bubble size represents the number of recovered isolates. Supplements consisted of sodium oleate, sodium lauryl sulfate, and bacitracin.

**Table 1:** Comparison of taxonomic recovery across isolation strategies and culture media conditions.

	Medium	No. of isolates	No. of non-redundant species	Taxonomic yield per screened isolate (%)
Method 1	TSA, supplements, sheep blood	49	7	14.3
Method 2	TSA	34	14	41.2
	TSA, supplements	67	12	17.9
	TSA, supplements, sheep blood	128	6	4.7
Conventional method	TSA, supplements, sheep blood	97	4	4.1

Method 1, direct-plating strategy; Method 2, enrichment strategy. Supplements consisted of sodium oleate (0.15%, w/v), sodium lauryl sulfate (0.01%, w/v), and bacitracin (50 mg/L); sheep blood was added at 5% (v/v). Taxonomic yield per screened isolate (%) was calculated as (number of non-redundant species / number of isolates) × 100. A total of 375 isolates were analyzed across all cultivation conditions.

## References

[1] Li W, Liang H, Lin X, Hu T, Wu Z, He W (2023). A catalog of bacterial reference genomes from cultivated human oral bacteria. NPJ Biofilms Microbiomes.

[2] Sizova MV, Hohmann T, Hazen A, Paster BJ, Halem SR, Murphy CM (2012). New approaches for isolation of previously uncultivated oral bacteria. Appl. Environ. Microbiol..

[3] Fleming E, Pabst V, Scholar Z, Xiong R, Voigt AY, Zhou W (2021). Cultivation of common bacterial species and strains from human skin, oral, and gut microbiota. BMC Microbiol..

[4] Müller S, Nebe-von-Caron G (2010). Functional single-cell analyses: flow cytometry and cell sorting of microbial populations and communities. FEMS Microbiol. Rev..

[5] Rappé MS, Connon SA, Vergin KL, Giovannoni SJ (2002). Cultivation of the ubiquitous SAR11 marine bacterioplankton clade. Nature.

[6] Zengler K, Toledo G, Rappe M, Elkins J, Mathur EJ, Short JM (2002). Cultivating the uncultured. Proc. Natl. Acad. Sci. USA.

[7] Moragues T, Arguijo D, Beneyton T, Modavi C, Simutis K, Abate AR (2023). Droplet-based microfluidics. Nat. Rev. Methods Primers.

[8] Watterson WJ, Tanyeri M, Watson AR, Cham CM, Shan Y, Chang EB (2020). Droplet-based high-throughput cultivation for accurate screening of antibiotic resistant gut microbes. Elife.

[9] Yin J, Chen X, Li X, Kang G, Wang P, Song Y (2022). A droplet-based microfluidic approach to isolating functional bacteria from gut microbiota. Front. Cell. Infect. Microbiol..

[10] Collins DJ, Neild A, deMello A, Liu AQ, Ai Y (2015). The Poisson distribution and beyond: methods for microfluidic droplet production and single cell encapsulation. Lab Chip.

[11] Lu H, Caen O, Vrignon J, Zonta E, El Harrak Z, Nizard P (2017). High throughput single cell counting in droplet-based microfluidics. Sci. Rep..

[12] Dong L, Chen DW, Liu SJ, Du W (2016). Automated chemotactic sorting and single-cell cultivation of microbes using droplet microfluidics. Sci. Rep..

[13] Zhang Q, Wang T, Zhou Q, Zhang P, Gong Y, Gou H (2017). Development of a facile droplet-based single-cell isolation platform for cultivation and genomic analysis in microorganisms. Sci. Rep..

[14] Staskiewicz K, Dabrowska-Zawada M, Kozon L, Olszewska Z, Drewniak L, Kaminski TS (2024). Droplet microfluidic system for high throughput and passive selection of bacteria producing biosurfactants. Lab Chip.

[15] Meng Y, Li S, Zhang C, Zheng H (2022). Strain-level profiling with picodroplet microfluidic cultivation reveals host-specific adaption of honeybee gut symbionts. Microbiome.

[16] Newman MG, Sutter VL, Pickett MJ, Blachman U, Greenwood JR, Grinenko V (1979). Detection, identification, and comparison of *Capnocytophaga*, *Bacteroidesochraceus*, and DF-1. J Clin Microbiol..

[17] Fujise O, Chen W, Rich S, Chen C (2004). Clonal diversity and stability of subgingival *Eikenella corrodens*. J. Clin. Microbiol..

[18] Malani AN, Aronoff DM, Bradley SF, Kauffman CA (2006). *Cardiobacterium hominis* endocarditis: Two cases and a review of the literature. Eur. J. Clin. Microbiol. Infect. Dis..

