# Supplementary material for: Reduced-Complexity Cultivation of Human Oral Anaerobic Bacteria Using a Microdroplet-Based Culturomics Platform
Source: J Microbiol Biotechnol. 2026 Oct 8;36:e2607042. doi: 10.4014/jmb.2607.07042 (PMC13686801; doi:10.4014/jmb.2607.07042)
Supplement: Supplementary file 1 [file jmb-36-e2607042-supple.pdf]

J. Microbiol. Biotechnol. 2026. 36: e2607042

https://doi.org/10.4014/jmb.2607.07042

Supplementary Tables

Table S1. Bacterial isolates recovered across different isolation methods and media compositions.

Supplements include sodium oleate (0.15%, w/v), sodium lauryl sulfate (0.01%, w/v), and bacitracin (50 mg/L). Sheep blood was supplemented at 5% (v/v). Taxa are listed with the corresponding number of isolates obtained under each condition.

Isolation method	Medium	Identification	No. of isolates
Method 1	TSA, supplements, sheep blood	*Capnocytophaga* sp.	1
		Cardiobacterium hominis	1
		Staphylococcus epidermidis	3
		Veillonella rogosae	33
		Veillonella parvula	2
		Veillonella hominis	8
		Veillonella dispar	1
Method 2	TSA	*Actinobaculum timonae*	1
		Actinomyces gerencseriae	4
		Actinomyces naeslundii	1
		Actinomyces oris	2
		Fusobacterium animalis	2
		Lancefieldella rimae	1
		Parvimonas micra	5
		Schaalia odontolytica	3
		Streptococcus constellatus subsp. *constellatus*	1
		Streptococcus constellatus subsp. *viborgensis*	1
		Streptococcus intermedius	1
		Streptococcus parasanguinis	3
		Streptococcus sanguinis	5
		Veillonella hominis	4
	TSA, supplements	*Campylobacter concisus*	7
		Fusobacterium canifelinum	1

		Fusobacterium hwasookii	6
		Fusobacterium polymorphum	11
		Fusobacterium animalis	1
		Klebsiella pneumoniae subsp. *ozaenae*	2
		Staphylococcus epidermidis	1
		Veillonella dispar	19
		Veillonella hominis	2
		Veillonella rogosae	15
		Veillonella tobetsuensis	1
		Veillonella parvula	1
	TSA, supplements, sheep blood	*Eikenella corrodens*	2
		Fusobacterium hwasookii	28
		Fusobacterium polymorphum	18
		Veillonella dispar	77
		Fusobacterium periodonticum	1
		Fusobacterium pseudoperiodonticum	2
Conventional method	TSA, supplements, sheep blood	*Campylobacter concisus*	8
		Fusobacterium periodonticum	25
		Veillonella hominis	7
		Veillonella rogosae	57
**Total number of isolates**			**375**

Table S2. Distribution of the number of bacterial species identified per positive growth well in recipient 96-well plates across four isolation trials.

Values represent the distribution of wells containing 1 to 4 distinct bacterial species, along with the total number of positive wells analyzed and the calculated mean species per well.

No. of species per well	Total
1	88
2	63
3	6
4	3
No. of wells	160
Mean no. of species per well	1.53

Table S3. List of 29 non-redundant bacterial species recovered in this study.

Species were identified via 16S rRNA gene sequencing of pure isolates obtained through the microdroplet platform and conventional culture.

No.	Species
1	*Actinobaculum timonae*
2	*Actinomyces gerencseriae*
3	*Actinomyces naeslundii*
4	*Actinomyces oris*
5	*Campylobacter concisus*
6	*Capnocytophaga* sp.
7	*Cardiobacterium hominis*
8	*Eikenella corrodens*
9	*Fusobacterium animalis*
10	*Fusobacterium canifelinum*
11	*Fusobacterium hwasookii*
12	*Fusobacterium periodonticum*
13	*Fusobacterium pseudoperiodonticum*
14	*Fusobacterium polymorphum*
15	*Klebsiella pneumoniae* subsp. *ozaenae*
16	*Lancefieldella rimae*
17	*Parvimonas micra*
18	*Schaalia odontolytica*
19	*Staphylococcus epidermidis*
20	*Streptococcus constellatus* subsp. *constellatus*
21	*Streptococcus constellatus* subsp. v*iborgensis*
22	*Streptococcus intermedius*
23	*Streptococcus parasanguinis*
24	*Streptococcus sanguinis*
25	*Veillonella dispar*
26	*Veillonella hominis*
27	*Veillonella parvula*
28	*Veillonella rogosae*
29	*Veillonella tobetsuensis*
